# Antioxidant, Anti-Inflammatory, and Metabolic Properties of Tocopherols and Tocotrienols: Clinical Implications for Vitamin E Supplementation in Diabetic Kidney Disease

**DOI:** 10.3390/ijms20205101

**Published:** 2019-10-15

**Authors:** Angelo Di Vincenzo, Claudio Tana, Hamza El Hadi, Claudio Pagano, Roberto Vettor, Marco Rossato

**Affiliations:** 1 Department of Medicine—DIMED, Clinica Medica 3, Center for the Study and Integrated Management of Obesity, University-Hospital of Padova, 35100 Padova, Italy; divincenzoang@gmail.com (A.D.V.); dr.hamza.elhadi@gmail.com (H.E.H.); claudio.pagano@unipd.it (C.P.); roberto.vettor@unipd.it (R.V.); 2 Internal Medicine and Critical Subacute Care Unit, Medicine Geriatric-Rehabilitation Department, and Department of Medicine and Surgery, University-Hospital of Parma, 43126 Parma, Italy; ctana@ao.pr.it; 3 Department of Medicine, Klinikum Rheine, 48431 Rheine, Germany

**Keywords:** vitamin E, diabetes, nephropathy, antioxidant, inflammation

## Abstract

Diabetes mellitus is a metabolic disorder characterized by the development of vascular complications associated with high morbidity and mortality and the consequent relevant costs for the public health systems. Diabetic kidney disease is one of these complications that represent the main cause of end-stage renal disease in Western countries. Hyperglycemia, inflammation, and oxidative stress contribute to its physiopathology, and several investigations have been performed to evaluate the role of antioxidant supplementation as a complementary approach for the prevention and control of diabetes and associated disturbances. Vitamin E compounds, including different types of tocopherols and tocotrienols, have been considered as a treatment to tackle major cardiovascular outcomes in diabetic subjects, but often with conflicting or even negative results. However, their effects on diabetic nephropathy are even less clear, despite several intervention studies that showed the improvement of renal parameters after supplementation in patients with diabetic kidney disease. Then we performed a review of the literature about the role of vitamin E supplementation on diabetic nephropathy, also describing the underlying antioxidant, anti-inflammatory, and metabolic mechanisms to evaluate the possible use of tocopherols and tocotrienols in clinical practice.

## 1. Introduction

In the light of several epidemiological and observational studies demonstrating the beneficial effects, vitamin E supplementation was historically considered as a possible treatment for slowing progression or even prevention of atherosclerotic cardiovascular disease (ASCVD). The prevalence of ASCVD is increasing because of the diffusion of conditions such as obesity and diabetes. According to the lipid hypothesis, emphasizing the role of low density lipoprotein (LDL) oxidation in the development of vascular plaque, the use of vitamin E, a natural antioxidant, was considered in both primary and secondary prevention. Thus, several large scale intervention trials were performed in the early part of the 21^st^ century to confirm the expected benefits of supplementation in patients with risk factors and both with or without clinical manifestations of ASCVD. Surprisingly, the majority of these studies showed negative results [1,2,3] and, consequently, the initial interest in this research area has dramatically decreased.

However, data from these trials have been debated for a long time by the experts, identifying some limitations in these studies’ designs to explain the unexpected results. The objections to these studies were represented by the dosage of supplementation, because most of the trials were performed using a low concentration mixture (usually corresponding to about 400 IU/die of α-tocopherol), and by the different composition of the mixture, which often included other molecules in addition to vitamin E. Furthermore, it has to be considered that vitamin E compounds include different lipid-soluble stereoisomers (α-tocopherol, β-tocopherol, γ-tocopherol, δ-tocopherol, and α-tocotrienol, β-tocotrienol, γ-tocotrienol, δ-tocotrienol) showing different biological properties, while the above-mentioned studies were performed using mainly α-tocopherols supplements. Thus, the lack of γ-tocotrienol (presenting the larger metabolic and cardiovascular effects among the above-mentioned stereoisomers) in these preparations has been proposed as an additional explanation for the negative results [4]. Finally, the heterogeneity of the considered populations (different age, sex, stage of atherosclerosis, comorbidities, smoking status) could further justify the variability of the results. Noteworthy, the current opinion disagrees with indiscriminate micronutrient supplementation, promoting a more tailored approach in more selected populations [5].

With regard to the role of tocopherols and tocotrienols in chronic diseases, promising results have been observed with supplementation in diabetic patients with renal abnormalities. Diabetic kidney disease (DKD) is a major complication of diabetes, characterized by a complex physiopathology with a relevant contribution of an imbalanced oxidant/antioxidant system. In this sense, a possible beneficial effect of antioxidant supplementation has been suggested. Despite a sub-study from one of the above-mentioned trials, the MICRO-HOPE trial failed to demonstrate a positive effect of vitamin E intervention on the microvascular outcomes [3]. On the contrary, other evidence showed its efficacy on the parameters of renal function.

Tocotrienols and tocopherols are known to present complex biological activity, and accumulating data confirm both the vascular and metabolic effects, suggesting a possible role for the prevention and the control not only of cardio-metabolic abnormalities but also renal abnormalities commonly observed in obesity and diabetes. Then, we performed a review of literature about the protective mechanisms of vitamin E in diabetes, and also about the clinical studies evaluating the effects of supplementation on the development and progression of DKD, evaluating its possible use in clinical practice.

## 2. Diabetes, Oxidative Stress, and Inflammation

### 2.1. Diabetic Kidney Disease

Diabetes, and in particular type 2 diabetes, remains one of the most relevant diseases of the modern era. The prevalence, as for obesity and metabolic syndrome, is increasing because of the spread of the western lifestyle worldwide. The burden of disease is related to the development of associated comorbidities, in particular ASCVD, leading to high costs for health systems because of hospitalization for acute complications such as acute myocardial infarction, or interventional procedures such as percutaneous coronary intervention, coronary artery bypass graft, lower limb amputation, and dialytic therapy [6]. Furthermore, diabetes is one of the main causes of blindness in adults. Vascular abnormalities occurring in diabetic patients are the main determinant of reduced life expectancy and are usually classified according to the vascular district affected (large blood vessels or small blood vessels) in macro- and micro-vascular complications. Clinical manifestations of diabetic angiopathy are mainly represented by stroke, coronary artery disease, peripheral artery disease, retinopathy, neuropathy, and nephropathy.

Diabetic nephropathy is a frequent microvascular complication observed in 20–40% of subjects with type 1 and type 2 diabetes, which actually represents the main cause of chronic kidney disease in Western countries. Histological abnormalities range from minimal alterations, such as glomerular membrane thickening, to severe glomerulosclerosis [7]. However, the underlying pathological mechanisms have not been completely clarified, probably involving multiple biochemical, anatomical, and functional alterations, leading to impaired glomerular filtration (glomerular thickening, podocytes processes regression) till to the development of the proteinuria. This late phase is usually preceded by a period of lower degree of proteinuria, also called *microalbuminuria* (an albumin excretion ranging from 30 to 299 mg/24 h), which has to be screened in all diabetic patients at the time of diagnosis to prevent overt nephropathy. The development of proteinuria represents the main predictor of mortality in diabetic subjects, and DKD is associated with a worse prognosis with respect to other causes of end-stage renal disease (ESRD) [8]. Thus, an intensive strategy for the optimal control of diabetes and the associated risk factors for nephropathy, in particular, arterial hypertension, is recommended in patients with microalbuminuria for preventing the progression to *macroalbuminuria*. Blood pressure and renal function are, in fact, strictly interconnected, and the development of kidney disease may promote arterial hypertension in a vicious circle in which the increase in blood pressure accelerates the progression of renal abnormalities, while the impaired renal function makes hypertension more resistant to pharmacological treatment. In this regard, the renin-angiotensin-aldosterone system-inhibiting drugs, acting both on systemic blood pressure levels and renal mechanisms of proteinuria, are usually preferred for hypertensive patients with diabetes in clinical practice.

The role of vitamin E supplementation in the management of DKD remains to be fully clarified. While in vitro studies demonstrated metabolic effects of antioxidant supplementation, in particular regarding insulin biology and activity [9,10], clinical studies are far from well establishing the mechanisms by which supplementation can be useful. Undoubtedly, the renal protection of these compounds moves primarily from their anti-oxidant and anti-inflammatory potential.

### 2.2. Oxidative Stress

Long-term hyperglycemia is considered the main factor responsible for the initiation of tissue and vascular abnormalities, with microvascular complications taking about 15 years to develop from the onset of diabetes. However, the mechanisms by which high blood glucose levels may contribute to the development of diabetic microangiopathy and, in particular, of DKD, remain unclear. The role of oxidative stress has been hypothesized and evaluated in microvascular disease, providing a deeper knowledge in the pathogenesis of diabetic complications.

Prolonged cellular exposure to high blood glucose levels is associated with an overproduction of reactive oxygen species (ROS) such as superoxide, hydrogen peroxide, and nitric oxide, leading to a reduction of natural endogenous (such as glutathione and superoxide dismutase) and exogenous (such as vitamin E) antioxidants. Intracellular high glucose levels, with the consequent increase in electron donors from the Kreb’s cycle, cause an increased potential at the inner mitochondrial membrane, which is responsible for the ROS excess [11]. Hyperglycemia-induced ROS overproduction leads to microvascular damage through different mechanisms, such as the increased formation of advanced glycation end products (AGEs), overexpression of relative receptors, and activation of polyol, protein kinase C (PKC), and nuclear factor κB (NFκB) pathways. PKC and NFκB activation lead to abnormal endothelial and vascular cell activity; in particular, PKC seems upregulated in renal glomeruli in diabetes, and inhibitors of PKC such as ruboxistaurin have been demonstrated to promote positive effects on albuminuria and glomerular filtration rate [12].

In DKD, the oxidative stress induces cellular apoptosis, glomerular distortion, and the regression of foot processes of podocytes, with consequent loss of integrity of the glomerular barrier. These abnormalities overlap other vascular alterations associated with persistently high levels of ROS. ROS overproduction impairs endothelial-derived nitric oxide synthase (e-NOS) activity [13], and, in addition, damaged endothelial cells also became able to release pro-coagulant molecules, such as von Willebrand factor (vWF), plasminogen activator inhibitor-1 (PAI-1) and thromboxane A2, further accounting for the microvascular impairment. However, antioxidant supplementation, in particular, γ-tocopherol, has been demonstrated to contrast hyperglycemia-impaired endothelial function [14].

### 2.3. Pro-Inflammatory Status

Along with pro-oxidant status, inflammatory and also pro-fibrotic signals promote renal injury. In addition to intravascular damage, inflammation in DKD is also associated with the infiltration of the extracellular matrix by inflammatory cells, fibrosis, and mesangial expansion, which are responsible for glomerular distortion and renal hemodynamic changes.

AGEs, which are proteins modified through a non-enzymatic reaction with glucose, are able to induce the inflammatory cascade interacting with the cell surface receptors (receptors for advanced glycation end product, or RAGEs) [15]. RAGEs are expressed on different cell types, not only endothelial cells but also fibroblasts, monocytes, and macrophages; after the interaction with their ligands, modulate several intracellular pathways such as NFκB, resulting in the transcription of pro-inflammatory genes. In fact, RAGEs activation leads to the expression of cytokines such as IL-1, IL-6, and TNF-α, and also vascular cell adhesion molecule-1 (VCAM-1) and intercellular adhesion molecule-1 (ICAM-1) [16]. The production of these molecules contributes to the development of diabetic angiopathy inducing interaction between platelets, immune, and endothelial cells, and further promoting the production of vWF, PAI-1, and other pro-coagulant molecules.

In addition, monocytes and macrophages infiltration have a key role in the development of mesangial abnormalities underlying DKD, which favors fibroblast activation and increases extracellular matrix deposition. Higher levels of monocyte chemoattractant protein-1 (MCP-1), a chemokine involved in the recruitment of the inflammatory cells in the peripheral tissues, have been demonstrated in subjects with DKD respect to controls and, interestingly, the supplementation with vitamin E has been associated with a reduction of MCP-1 plasma levels [17], confirming a possible positive effect of vitamin E on inflammation related to diabetes.

The oxidation of lipoproteins is considered as the main determinant of atherosclerosis and represents the overlap between inflammation and oxidative stress, promoting micro- and macro-vascular complications of diabetes. In the condition of impaired endogenous anti-oxidant system, circulating LDLs are oxidized and accumulated in the vascular matrix. These molecules present high pro-inflammatory activity, which further increases the production of inflammatory mediators. After the expression of adhesion molecules, macrophages transmigrate in the vascular wall, phagocytizing the oxidized LDLs, and transforming into foam cells [18]. The persistence of local inflammation along the tunica intima leads to the formation of atheroma.

**Figure 1:**
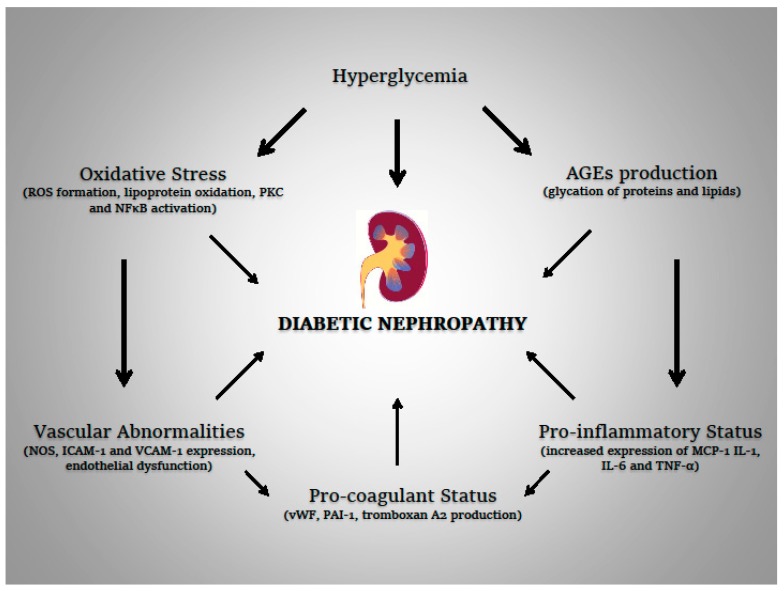
The physiopathological mechanisms involved in the development of diabetic nephropathy are reported. The arrows define the specific physiopatological correlations according to the evidence. Legend: ROS = reactive oxygen species; AGEs = advanced glycation end products; MCP-1 = monocyte chemoattractant protein-1; vWF = von Willebrand factor; PAI-1 = plasminogen activator inhibitor-1; NOS = nitric oxide synthase; ICAM-1 = intercellular adhesion molecule-1; VCAM 1 = vascular cell adhesion molecule-1.

Then, diabetic angiopathy in diabetes is consequent to multiple pathologic processes involving both antioxidant and inflammatory system (the physiopathological mechanisms involved in the development of diabetic nephropathy are reported in Figure 1); a better knowledge of the involved mechanisms could be of help in identifying new therapeutic strategies, and further efforts in this direction are needed.

## 3. Diabetic Nephropathy and Vitamin E

Despite hyperglycemia being the main physiopathological feature of diabetes and related complications, the rates of the cardiovascular manifestations seem related not with metabolic control alone, and, in fact, cardiovascular complications and mortality in diabetic subjects are not predictable only on the basis of the intensity of glucose-lowering therapy. In this sense, alternative approaches have been suggested for the prevention of these complications besides the primary prevention with antiplatelet therapy: Promoting the protection of endothelial cells, reducing inflammation, increasing antioxidant molecules levels, and fighting mechanisms of injury such as AGE and ROS production, which could reduce the burden of complications even in a condition of non-optimal diabetic control.

The role of vitamin E supplementation for the prevention and slowing the progression of metabolic diseases and associated complications is still controversial. Vitamin E status in subjects with both pre-diabetes and diabetes seems to positively modulate glycemic control irrespective of the anti-oxidant activity. Some reports even suggested that the low levels of vitamin E lead to an increased risk of developing type 2 diabetes. Antioxidants supplementation has been extensively evaluated in diabetic subjects, and several studies have described the beneficial effects on glucose metabolism [19,20], with some reports also confirming these results in gestational diabetes: In particular, a recent RCT showed that vitamin E co-administered with magnesium during pregnancy resulted in the amelioration of several metabolic parameters such as fasting plasma glucose, total cholesterol, triglycerides, LDL-cholesterol, and in an improvement of the homeostasis model assessment

(HOMA) index [21]. Thus, vitamin E seems to present beneficial effects on dyslipidemia, and it is relevant to note that the possible role of tocopherols and tocotrienols on the lipid profile might have an additional role in subjects with advanced nephropathy who are at higher cardiovascular risk respect to patients without renal disease. However, these results have not been reproduced in other studies.

Antioxidant supplementation in subjects with nephropathy has shown promising results in slowing progression of atherosclerosis: Even a short-term, low dose, vitamin E supplementation (almost 300 mg/die) seems to improve, in patients with ESRD undergoing hemodialysis, the flow-mediated arterial dilation (FMD), an indirect method to assess the endothelial function [22]. These vascular effects could mediate the protective role of tocopherols and tocotrienols on DKD. A recent meta-analysis confirmed these effects of vitamin E supplementation on FMD in patients with diabetic nephropathy (95% CI, 0.23, 0.72, *p* = 0.0001), irrespective of daily dosages (ranging from 300 to 1800 mg/day in the different studies) [23]. Noteworthy, the authors reported that the subgroups analysis suggested a more accurate identification of patients suitable for supplementation in order to provide a greater benefit.

Several antioxidant molecules have been specifically evaluated for diabetic nephropathy, such as AGE inhibitors, PKC inhibitors, or transketolase activators. Furthermore, considering the role of vascular tone (in particular of afferent arteriola) in the development of renal abnormalities, drugs have been proposed in the management involving some pathways such as e-NOS, endothelin-1, and vascular–endothelial growth factor. Supplementation with vitamin E and other antioxidants such as vitamin C and lipoic acid was considered with great interest because of the easy and large availability of these molecules, despite several aspects regarding their effects on DKD and the underlying physiopathological mechanisms still remaining unclear.

### 3.1. Vitamin E and Diabetic Nephropathy: Clinical Evidence

A meta-analysis of RCTs evaluating the role of antioxidant supplementation (vitamin E and other molecules such as vitamin A, vitamin C and selenium, alone or in combination) on DKD confirmed the positive effects of vitamin E in ameliorating parameters of renal function, in particular urinary albumin excretion (SMD −0.33; −0.61, −0.04) [24]. However, due to the heterogeneity of the reported outcomes from each study and the different types and durations of the antioxidant treatment, these results cannot be generalized, and the expected effects of supplementation are probably dependent on the patients’ characteristics.

The beneficial effects of antioxidant supplementation on diabetic nephropathy with micro or macroalbuminuria were demonstrated in the past [25], and recent studies confirmed the potential application of tocotrienols and tocopherols in patients with diabetic nephropathy [26]. In addition, vitamin E supplementation in patients with ESRD and pre-existent cardiovascular disease seems to reduce even the risk of fatal and non-fatal myocardial infarction [27], confirming the concomitant renal and systemic beneficial effects of vitamin E supplementation acting on multiple pathways, such as inflammation, vascular health, and oxidative stress. In this regard, some authors suggested a metabolic effect of tocopherols supplementation influencing the cardiometabolic risk factors above the antioxidative activity. In a recent small RCT, Aghadavod et al. showed that high-dose vitamin E supplementation was associated with a significant amelioration of plasma lipid profile, in particular with a reduction of LDL- and an increase of HDL-cholesterol [28]. Other studies reported a beneficial effect after vitamin E supplementation not only on the lipid profile but also on post-prandial glycemia and blood pressure in diabetic subjects [29]. Even if the mechanisms justifying these effects remain unclear, the possible modulation of lipid and glucose metabolism could delay the progression of both diabetic complications and cardiovascular manifestations, thus further supporting the role of antioxidant treatment in these patients.

Genetic susceptibility largely contributes to the development of diabetic complications, and several polymorphisms in loci counter-regulating the antioxidant systems have been associated with DKD; then genetics could also influence the clinical efficacy of vitamin E supplementation. The haptoglobin 2-2 genotype (Hp 2-2) is characterized by a reduced response to oxidative stress and is associated with an increased prevalence of DKD, a more rapid progression to end-stage renal disease, and more pronounced histological abnormalities. Interestingly, in pre-clinical studies, vitamin E showed more pronounced effects in models with Hp 2-2 genotype with respect to Hp 1-1 genotype [30].

With respect to type 1 diabetes, increased levels of oxidation markers are correlated with the development of early nephropathy and kidney abnormalities such as glomerular hyperfiltration [31], and vitamin E supplementation seems to be ineffective if started after the development of microalbuminuria [32]. Probably, in these patients, antioxidant supplementation could have a time-dependent efficacy, with limited effects in the more advanced stages of the disease. To this regard, a previous randomized controlled trials (RCTs) showed a significant improvement of creatinine clearance in association with a significant amelioration of retinal blood flow with high dose of vitamin E (α-tocopherol, 1800 IU/day) in patients with a diagnosis of type 1 diabetes from less than 10 years and without microalbuminuria [33], confirming the utility of early supplementation in these subjects.

A summary of studies evaluating the effects of antioxidant supplementation in diabetic subjects with nephropathy is reported in Table 1.

### 3.2. Vitamin E and Diabetic Nephropathy: Antioxidants and Anti-Inflammatory Mechanisms

The mechanisms by which vitamin E could modulate the onset or the progression of kidney abnormalities in DKD are far from being fully elucidated. An impaired antioxidant status, along with the reduction of vitamin E concentration are well known to be associated with the development of diabetic complications [36], then several studies proposed the role of antioxidant supplementation in preventing macroalbuminuria and overt nephropathy in the early phase of microalbuminuria. Likely, vitamin E influences kidney function in DKD through different biochemical pathways and acting on different cell targets, such as podocytes, endothelial cells, and mesangial cells [37]. However, considering observations about the protective role of vitamin E against nephrotoxic agents, the mechanisms of renal protection are probably even more complex. In fact, vitamin E compounds have also been tested for the prevention of contrast-induced nephropathy, showing beneficial effects on this serious complication of radiological examinations [38]. Radiological contrast agents present a complex activity inducing renal damage: Their cytotoxic effects on endothelial and tubular cells are modulated by ischemic damage, vasoconstriction with a concomitant reduced vasorelaxant response, medullary hypoxia, and then increased oxidative stress, with the reduction of nitric oxide and the overproduction of ROS. Vitamin E supplementation seems to prevent these abnormalities, as observed in patients with chronic kidney disease undergoing coronary angiography [34].

A recent elegant study contributed to unraveling the mechanisms by which vitamin E improves renal function: The authors demonstrated in animal models that the amelioration of DKD induced by vitamin E supplementation was mediated by the activation of diacylglycerol kinase, an enzyme that, by reducing circulating levels of diacylglycerol, prevented abnormal activation of PKC and the regression of podocytes [39]. A previous study showed that tocotrienol supplementation acted on DKD through both the inhibition of the NFκB pathway and the reduction of the inflammatory response [40]. To this respect, as demonstrated by a recent RCT, high dose vitamin E supplementation was associated with a significant reduction of TNF-α, MMP-2 and MMP-9, irrespective of glycemic control [35]. Furthermore, tocopherols supplementation has also been associated with the reduction of serum levels of pro-inflammatory mediators increased after lithotripsy, suggesting a specific role on inflammation related to renal and urologic affections [41]. The majority of these data regarding the action of vitamin E in DKD are available from pre-clinical studies, largely confirming the effects of supplementation on reducing pro-inflammatory mediators, oxidation markers, and on improving clinical parameters of renal function [42,43], and kidney histology [39]. Recently, high dose vitamin E supplementation was shown to reduce both renal interstitial fibrosis and tubular epithelial cells apoptosis [44], suggesting a novel physiopathological mechanism for DKD and a possible therapeutic target. Furthermore, in animal models of diabetes, supplementation of vitamin E also seems to have positive effects on renal vasculature [45], but more studies, and in particular clinical studies, are needed to support this initial evidence.

However, some beneficial effects of vitamin E compounds, in particular, α-tocopherol, may also be independent from an antioxidative or combined anti-inflammatory capacity. In fact, according to some observations, α-tocopherol seems to exert a greater inhibitory activity on PKC than β-tocopherol, probably interacting with a peculiar enzyme isoform. Furthermore, despite a similar anti-inflammatory activity, it presented a wider activity on reducing pro-inflammatory molecules such as IL-β. This could be explained by an additional mechanism of α-tocopherol such as the inhibition of the lipoxygenase pathway [46]. Similarly, α-tocopherol also presented a peculiar activity on diacylglycerol kinase, through an interaction with a specific enzyme subtype (α-diacylglycerol kinase), suggesting a further non-oxidative renal effect of α-tocopherol [47]. A different biological profile was also described for γ-tocopherol. In fact, despite their comparable anti-inflammatory activity, γ-tocopherol, with respect to α-tocopherol, was showed to present only a weak suppressive activity on scavenger receptor and activator protein 1 [48].

### 3.3. Vitamin E and Diabetic Nephropathy: Metabolic Mechanisms

Considering the results of some clinical studies, showing the amelioration of the lipid profile and a better glycemic control after tocopherols and tocotrienols supplementation, several hypotheses have been proposed to explain these data, and the possible modulation of adipose tissue functions by vitamin E has been suggested as a putative mechanism. Several reports showed that γ-tocotrienols have a relevant effect on lipids, reducing circulating levels of free fatty acids, triglycerides, and cholesterol through the modulation of the 3-hydroxy-3-methylglutaryl-CoA reductase. In addition, the supplementation reduces body weight and ameliorates body composition, reducing fat mass in animal models. Abnormalities of visceral adipocytes are usually considered as a determinant factor for the development of metabolic diseases such as type 2 diabetes, but also of obesity-related cardiovascular complications. In fact, adipose tissue is an active endocrine organ, influencing systemic homeostasis through the production of several mediators called adipocytokines, such as leptin, adiponectin, but also TNF-α, IL-6, and MCP-1, acting as hormones or chemo-cytokines. In conditions such as obesity and metabolic syndrome, these molecules are responsible for the induction of a systemic inflammatory state and insulin resistance. Vitamin E seems to modulate the activity of these molecules: The integrity of the antioxidant system leads to reduced circulating levels of leptin [49], while macronutrient supplementation including α-tocopherol seems to have a relevant influence on both leptin and adiponectin biology [50]. These metabolic consequences could further explain how tocopherols and tocotrienols supplementation exerts its positive effects on DKD. However, these mechanisms are not completely understood, and on the other hand, some reports have described even negative effects of antioxidant molecules on adipose tissue. In fact, despite its beneficial properties, a study observed how vitamin E could lead to a paradoxical worsening of mitochondrial dysfunction in adipocytes after an adrenergic stimulation via β_3_-receptor activation carried out to promote white adipose tissue browning [51]. Furthermore, vitamin E supplementation has sometimes shown no favorable metabolic effects when provided in the absence of oxidative stress. In physiological conditions, ROS production seems to be involved in the adipogenic process and insulin sensitivity, and then an impaired oxidative milieu might promote insulin resistance [52]. On the other hand, it has been demonstrated that vitamin E supplementation reduces the adipocytes size and adipose tissue macrophage infiltration in models of high-fat diet-induced obesity [53]. These effects may be partially explained by the induction of the expression of genes involved in adipocyte differentiation, in particular PPARγ and PGC-1α [54,55]. Furthermore, these experiments raised the intriguing hypothesis that vitamin E could be an activator of non-shivering thermogenesis. In particular, δ-tocopherols seem to be able to induce UCP-1 expression in adipocytes, and then they could activate the browning of white adipose tissue, thus further strengthening the positive metabolic effects of vitamin E on diabetes and then diabetic nephropathy development. However, further studies are needed to confirm this possible mechanism of action.

## 4. Conclusions

DKD is a relevant complication of diabetes, which is associated with a worse prognosis. Antioxidant treatment may be beneficial in association with the standard therapies, considering the role of inflammation and oxidative stress in its development. The complexity and heterogeneity of all the physiopathological mechanisms involved, as well as the different study designs, may account for the contradictory results obtained in the clinical trials testing vitamin E supplementation on vascular outcomes in diabetes. However, large evidence suggests a potential role for antioxidants in patients with diabetic nephropathy for slowing progression to the advanced stages, in particular, it may be beneficial in some categories such as patients with type 1 diabetes and without microalbuminuria, or Hp 2-2 genotype subjects. It is relevant to remember that supplementation is per se an additional approach, while a sweeping treatment with vitamin E is not recommended. On the contrary, a patient’s tailored supplementation with tocopherols and/or tocotrienols could be useful in some subsets of diabetic patients. Further studies will be helpful to confirm their application in clinical practice.

## Figures and Tables

**Table 1:** Studies evaluating the effects of vitamin E supplementation on diabetic patients with renal disease.

Authors	Year	Study Design	Population	Results
Mune et al. [22]	2018	Prospective	Diabetic and non-diabetic patients with ESRD	Amelioration of endothelial function in diabetic subjects
Bolignano et al. [24]	2017	Meta-analysis	Type 1 and type 2 diabetes with DKD	Reduction of albuminuria; no effects on GFR
Gaede et al. [25]	2001	RCT	Type 2 diabetes with DKD	Reduction of albuminuria
Tan et al. [26]	2018	RCT	Type 2 diabetes with DKD	Reduction of serum creatinine; no effects on proteinuria
Aghadavod et al. [28]	2018	RCT	Type 1 and type 2 diabetes with DKD	Positive effects on lipid profile; no evaluation on renal function
Baburao et al. [29]	2012	n-RCT	Type 1 and type 2 diabetes with or without DKD and/or other vascular complications	Positive effects on the development and progression of vascular complications
Giannini et al. [32]	2007	RCT	Type 1 diabetes with microalbuminuria	Positive effects on oxidative stress; no effects on albuminuria
Bursell et al. [33]	1999	RCT	Type 1 diabetes	Positive effects on creatinine clearance
Rezaei et al. [34]	2016	RCT	Diabetic and non-diabetic patients with chronic kidney disease undergoing contrast medium administration	Reduced incidence of contrast-medium induced kidney injury
Khatami et al. [35]	2016	RCT	Type 2 diabetes with DKD	Reduction of proteinuria, oxidative and inflammatory markers

## Abbreviations

ASCVD	Atherosclerotic cardiovascular disease
LDL	Low-density lipoprotein
IU	International Unit
DKD	Diabetic kidney disease
MICRO-HOPE	Microalbuminuria, Cardiovascular and Renal Outcomes–Heart Outcomes Prevention Evaluation
ESRD	End-stage renal disease
ROS	Reactive oxygen species
AGEs	Advanced glycation end products
PKC	Protein kinase C
NFκB	Nuclear factor κB
e-NOS	Endothelial-nitric oxide synthase
vWF	Von Willebrand factor
PAI-1	Plasminogen activator inhibitor-1
RAGEs	Receptors for advanced glycation end products
IL-1	Interleukin-1
IL-6	Interleukin-6
TNF-α	Tumor necrosis factor-α
VCAM-1	Vascular adhesion molecular-1
ICAM-1	Intercellular adhesion molecule-1
MCP-1	Monocyte chemoattractant protein-1
RCTs	Randomized controlled trials
HOMA	Homeostasis Model Assessment
FMD	Flow-mediated dilation
SMD	Standardized mean difference
Hp	Haptoglobin genotype
MMP	Matrix metalloproteinase
